# From prediction to practice: machine learning models and real-world interventions for magnetic resonance imaging appointment no-shows in a hospital setting

**DOI:** 10.1093/jamiaopen/ooag151

**Published:** 2026-08-04

**Authors:** Mark McMahon, Jonas Kluckert, Olivio F Donati, Nicolas Andres Perez Gonzalez, Laura Kinkead, Christian Blüthgen, Andreas Hötker, Thomas Frauenfelder, Michael Krauthammer

**Affiliations:** Department of Quantitative Biomedicine, University of Zürich, Zürich, 8006, Switzerland; Biomedical Informatics, University Hospital of Zürich, Zürich, 8091, Switzerland; Department of Quantitative Biomedicine, University of Zürich, Zürich, 8006, Switzerland; Biomedical Informatics, University Hospital of Zürich, Zürich, 8091, Switzerland; Institute for Diagnostic and Interventional Radiology, University Hospital Zurich, Zürich, 8091, Switzerland; Institute for Diagnostic and Interventional Radiology, University Hospital Zurich, Zürich, 8091, Switzerland; Department of Quantitative Biomedicine, University of Zürich, Zürich, 8006, Switzerland; Biomedical Informatics, University Hospital of Zürich, Zürich, 8091, Switzerland; Department of Quantitative Biomedicine, University of Zürich, Zürich, 8006, Switzerland; Biomedical Informatics, University Hospital of Zürich, Zürich, 8091, Switzerland; Institute for Diagnostic and Interventional Radiology, University Hospital Zurich, Zürich, 8091, Switzerland; Institute for Diagnostic and Interventional Radiology, University Hospital Zurich, Zürich, 8091, Switzerland; Institute for Diagnostic and Interventional Radiology, University Hospital Zurich, Zürich, 8091, Switzerland; Department of Quantitative Biomedicine, University of Zürich, Zürich, 8006, Switzerland; Biomedical Informatics, University Hospital of Zürich, Zürich, 8091, Switzerland

**Keywords:** machine learning, radiology, appointment no-shows, appointments and schedules, intervention study

## Abstract

**Objective:**

To develop a machine learning (ML) model to predict magnetic resonance imaging (MRI) appointment no-shows and test the effectiveness of a targeted phone call intervention in reducing the no-show rate.

**Materials and Methods:**

Outpatient MRI appointments from a university hospital (2015–2021) were used to train and compare Logistic Regression, Random Forest, and XGBoost models. The best-performing model underwent a 13-month prospective evaluation, followed by a 9-month intervention study where high-risk patients were randomized to a phone call reminder or a control group.

**Results:**

Our dataset included 38 141 appointments (16.6% no-show) in total. On a held-out validation dataset, XGBoost performed best (AUROC 0.65; AUPRC 0.29). In a prospective evaluation round (5882 appointments), performance decreased (AUROC 0.61; AUPRC 0.24), due in part to observed data drift in appointment types and reasons. The intervention study (274 patients called, 158 answered, 116 not reached; 460 control appointments) showed no overall change in no-show rate (no-show: 21.9% intervention vs 22.0% control; *P* = .48). Within the intervention group, answering the call was associated with lower no-show rates (18.4% of those reached vs 26.7% not reached).

**Discussion:**

Models identified high-risk patients, but translating predictions into attendance improvements proved challenging. Effectiveness was limited by data drift and intervention reach. Results suggest value in dual-prediction strategies (including potential intervention responsiveness) and continuous model monitoring.

**Conclusion:**

ML can identify likely no-shows, but reducing missed appointments requires robust, up-to-date models and interventions that reliably reach patients; more nuanced, targeted engagement may yield greater impact than phone calls alone.

## Background and significance

Missed appointments represent an ongoing challenge for the healthcare system. The problem has consistently been described as costly,^1–3^ and estimates place no-show rates in the order of 3%-31%.^3–7^ This is particularly significant for magnetic resonance imaging (MRI) examinations, where costs of non-utilization are substantial due to the high acquisition and operational costs. In addition to economic concerns, appointment no-shows contribute to worse health outcomes, lower overall quality of care,^8^ and delays in diagnosis and treatment, which in turn are reported to lead to increased morbidity and mortality.^4^^,^^9^

Machine learning (ML) has been explored as a tool for identifying patients at high risk of missing appointments.^10^^,^^11^ Multiple studies have attempted to characterize and model no-show behavior in the clinic,^4^^,^^5^ showing that features such as the day of the week and time of the day of the appointment, as well as previous attendance behavior and scheduling lead time correlate with no-show behavior.^2^^,^^4^ Additionally, patient characteristics such as age and socioeconomic status have also shown a correlation with no-show behavior.^4^ However, while much of the literature focuses on model development and validation, less attention has been given to the deployment of these models in clinical settings and evaluating the effectiveness of intervention strategies informed by prediction models. In recent years, studies aiming to apply such models in practice have become increasingly common,^12^ though their results have been mixed. Despite this growing interest, the effectiveness of data-driven interventions remains insufficiently explored.

**Figure 1.**
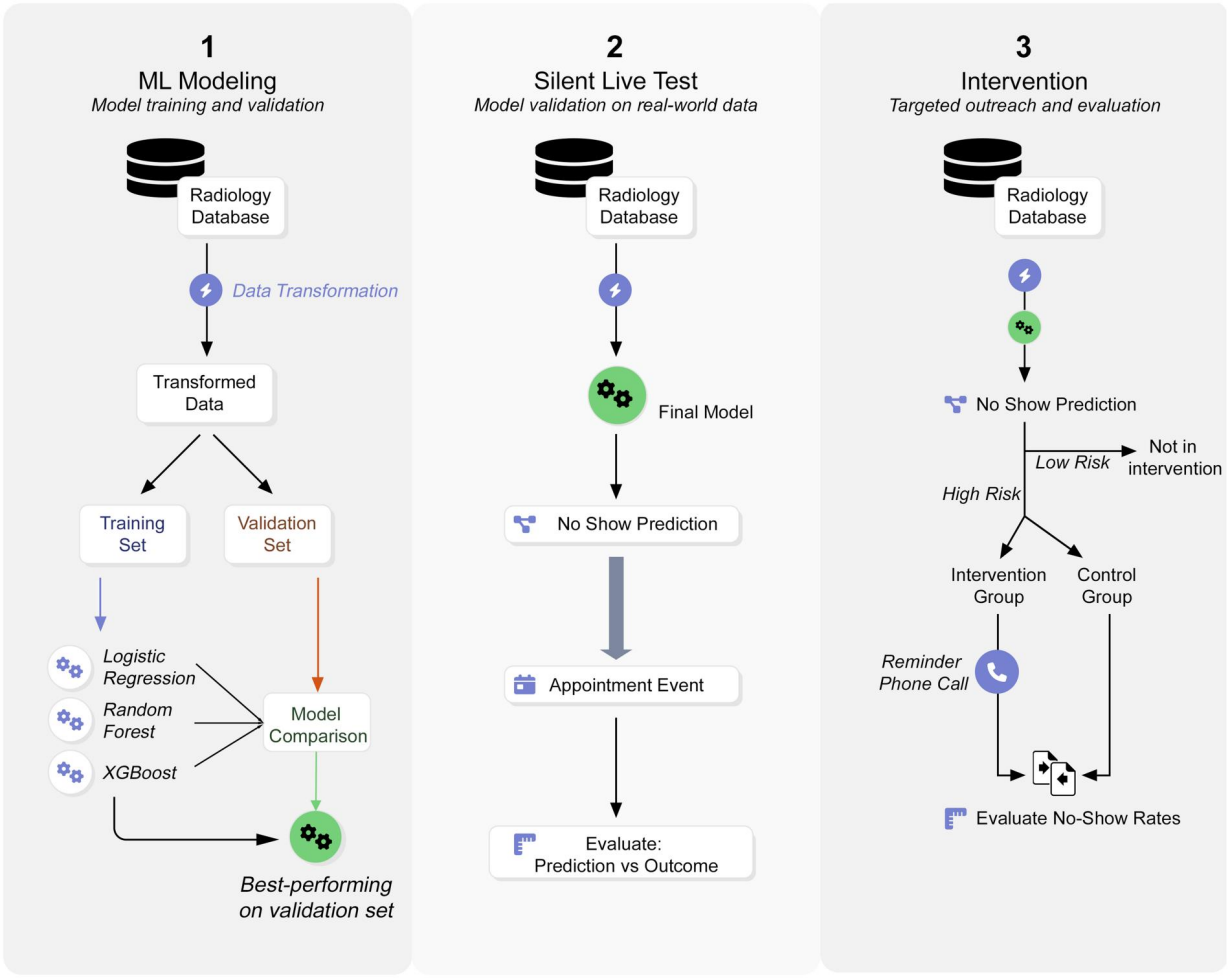
Experimental Framework. 1: Data Collection and Model Training: Raw data from the database was transformed and validated to robustly indicate show and no-show appointments. The transformed data was split into training and validation sets, and used train and compare three model set-ups. 2: To evaluate the best performing model prospectively, a ‘silent live test’ was conducted by prospectively predicting upcoming no-shows and monitoring model performance. 3. Following this, an Intervention-Control experiment was performed in an attempt to reduce the no-show rate.

This study aims to bridge this gap by developing and validating ML models that predict MRI appointment no-shows, and evaluating the real-world impact of such models through targeted interventions. Our approach consists of three key phases (Figure 1): (1) data collection and transformation, (2) model development and real-world validation to detect high-risk patients, and (3) an intervention study in which these patients received phone call reminders of their appointment. By combining predictive modeling with a real-life intervention, this study offers insights into the use of ML to address the issue of no-shows in healthcare, while revealing some of the practical challenges of real-world deployment.

## Methods

### Data and setting

This study, conducted in the radiology department at the University Hospital of Zurich, used data from 2015 to 2021 and included outpatient appointments from two MRI machines. These machines primarily handled body examinations, with occasional head appointments; most head appointments, however, were performed in other hospital departments. We obtained our data through the Research Data Service Center (RDSC) at the hospital, extracting appointment data from the Radiology Information System (RIS) database, and patient data from electronic medical records stored in the hospital’s clinical information system (KISIM). RIS data were structured as an “audit history”, with multiple entries per appointment, each corresponding to changes in the appointment status (eg, “requested,” “scheduled,” “completed”).

In this study, a no-show event is defined as a patient giving less than 2 days notice, or no notice at all, of nonattendance. This 2-day threshold reflects the fact that short-notice cancellations limit the scheduling staff’s ability to reassign the time slot, often resulting in machine idle time and under-utilization of resources.

### Data transformation

After extracting the ‘audit history’ data from RIS, we transformed these records into an appointment-level dataset with a show/no-show outcome label. Because this transformation was not predefined, we validated it by comparing a subset of the pipeline’s output against ground truth data collected manually from the hospital’s front-end system. Manual collection and labeling of a full dataset was not feasible, but the validated subset confirmed the accuracy of our transformation pipeline and supported the creation of a robust dataset for model training.

### Model features

During data transformation, we created the binary outcome variable (show/no-show), and a set of features derived from appointment-specific and patient-specific data. The available features are summarized in Table 1. These include appointment details such as the hour, day, and reason for the appointment; patient demographics such as age, sex, and occupation; historical patient-appointment behavior; and scheduling considerations, including the patient’s distance from the hospital and the number of days the appointment was scheduled in advance.

**Table 1.** Features used in the prediction model.

Feature	Description
Hour scheduled	Hour of the day the appointment is due to take place
Day of the week	Day of the week the appointment is due to take place
Month	Month of the year the appointment is due to take place
Sex	Patient sex (male or female)
Occupation status	Patient’s working status (eg, student, employed, retired, etc.)
Age	Patient age at time of appointment
Marital status	Patient’s marital status (married, single, divorced, unknown)
Distance to hospital	Patient’s travel distance to the hospital
Reason	The given reason for the appointment
Modality	Type of image modality used
# Previous appointments	Number of appointments the patient has previously had at the radiology department
Historic no-show rate	Percentage of previous no-shows for that patient
Days scheduled in advance	Days scheduled before appointment
Times rescheduled	Number of times the appointment was rescheduled

### Model building and evaluation

The transformed dataset was used to predict appointment outcomes—specifically, whether an appointment slot will result in a show or a no-show. We randomly split the dataset into training and validation sets, using an 80%-20% split. The training set was used for model experimentation, while the validation set was used as a hold-out set to evaluate our final model’s performance.

We tested three ML approaches—Logistic Regression, Random Forest, and XGBoost—based on their frequent use in no-show prediction research.^2^^,^^13–16^ For each model, we conducted 5-fold cross-validation on the training set, optimizing hyperparameters via Bayesian search within each cross-validation loop. The best performing model was determined by its average performance across the folds, and was then retrained on the entire training set and evaluated on the hold-out validation set. Finally, this configuration was then used to train a model using all the available data, which would then be used later for the silent live test (SLT) and the intervention.

#### Evaluation metrics

We evaluated model performance using standard classification metrics, specifically the area under the receiver operating characteristic curve (AUROC) and the area under the precision-recall curve (AUPRC). To improve model interpretability and transparency, we calculated SHapley Additive exPlanations (SHAP) values. SHAP values quantify how each feature contributes to the predicted outcome for individual appointments, providing insights into the impact of specific feature values on the model’s predictions. This approach helps to illustrate the factors influencing the model’s prediction-making process.

To assess the model’s applicability in a hospital setting, we introduced a “Top-N Precision” metric, where *N* represents the number of patients targeted for intervention each week. Patients were ranked by their predicted no-show probability, and the metric reflects the proportion of true no-shows among the top *N* ranked cases, compared against a baseline of random selection (≈0.17 in our data). For example, a Top-10 Precision of 0.4 would mean that, among the 10 patients predicted at highest risk in a given week, 4 actually missed their appointments. This metric helps evaluate the model’s effectiveness in identifying likely no-show patients, and *N* can be adjusted to tailor intervention goals and guide decisions on the optimal number of patients to target.

### Silent live test

To assess real-world model performance prior to intervention, we conducted a SLT, generating daily no-show predictions for upcoming appointments (3-5 business days ahead) using live data, previously unseen by the model. Predictions were compared with actual outcomes to evaluate the prospective accuracy of the model. The SLT also served as a dry run of the full intervention pipeline, including data extraction, transformation, prediction, and communication to staff, without requiring any active intervention.

### Intervention

An important aspect of this project was the intervention study, where we aimed to reduce the no-show rate at the radiology department using predictions from the best-performing model. In collaboration with the scheduling staff, we designed a study which targeted patients most at risk of no-showing, according to the model. Identified patients were randomly assigned to either an intervention group—receiving phone call reminders—or a control group receiving no reminders.

#### Study design and group assignment

**Figure 2.**
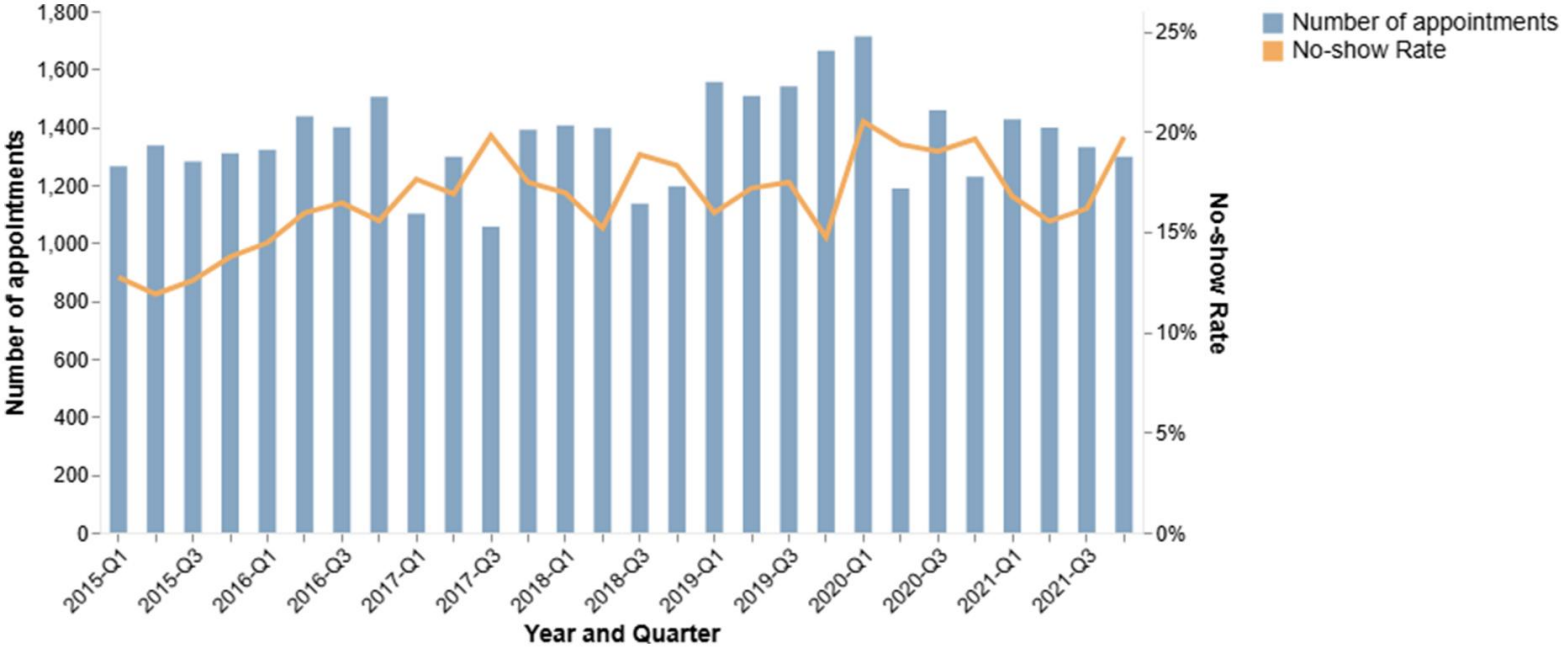
The number of appointments per quarter for the two MRI machines included in the study, along with the no-show rate.

Appointments with a predicted no-show probability above a certain threshold were considered for the study. This threshold was calculated, using historical model predictions, to yield approximately 10 phone calls to high-risk patients per week, while also including enough patients to build the control group. Rather than relying on historical no-show rates as a control, which varied over time (Figure 2), we collected control data during the intervention period. This ensured both groups were drawn from the same population, with similar risk levels, isolating the intervention effect.

On calling days, patients exceeding the predicted risk threshold were randomly split into an intervention group and a control group. On non-calling days, high-risk patients were assigned directly to the control group. If calls could not be made due to holidays or staff constraints, both intervention and control patients for that day were excluded from the study.

#### Execution

Phone calls were made on Mondays and Thursdays, contacting patients in the intervention group 3-5 business days before their appointments. This schedule ensured coverage of appointments throughout the week, while also minimizing additional workload for the scheduling staff. All calls were made by the same staff member, a scheduling coordinator in the radiology department, whose routine duties include patient contact. No formal script was used; the caller reminded patients of their upcoming appointment, and could answer routine questions. Call outcomes were simply recorded as “reached” (call answered and reminder delivered) or “not reached” (unanswered, no systematic retry), a deliberately lightweight intervention to minimize staff burden. Patients who answered typically confirmed their attendance, or occasionally rescheduled. Both outcomes were considered successful interventions, as they helped avoid potential no-shows.

#### Statistical tests

A power analysis was conducted to determine the required sample size, while ensuring at least 80% statistical power, to detect a reduction of one no-show per week in the intervention group compared to the control group. We used a *z*-test to compare the no-show rates between the control and intervention groups. This statistical test is appropriate for assessing the significance of differences in proportions—in this case, whether reminder calls significantly reduced no-show rates.

### Application user interface

To assess the feasibility of end-to-end automation, we developed a prototype front-end interface, designed for use by scheduling staff in the radiology department. The system collects appointment data, evaluates no-show risks, and presents the results to the scheduling staff with a daily appointment schedule, along with no-show risk levels for upcoming appointments. Usability and staff acceptance was evaluated using a simplified version of the Questionnaire for User Interaction Satisfaction (QUIS).^17^

### Ethical considerations

We received a waiver to collect and process the hospital appointment data via a clarification of responsibility request to the Cantonal Ethics Committee (BASEC Request Number: 2019-00890). All data records were anonymized, with patient names removed and identification numbers replaced by coded identifiers.

## Results

### Data transformation

The extracted “audit history” dataset contained 300 862 records of appointment status updates for 48 024 outpatient appointments and 28 428 patients, spanning from 2015 to 2021. In addition to this original dataset, we manually collected ground-truth records for 329 appointments, for use in refining our transformation pipeline. Of these manually collected appointments, 164 were used iteratively to refine our initial pipeline, while the remaining 165 served as a hold-out validation set. Initially, our pipeline showed discrepancies, achieving a reconstruction accuracy of only 59.0% (Jaccard Index). Through iterative refinements, we improved the reconstruction accuracy to 99.7%. The resulting pipeline could then be used on the full historical dataset, thus providing a robust, validated appointment dataset which closely aligns with the ground truth.

As a final data processing step we removed appointments which were scheduled on very short notice (less than 2 days, in line with the no-show definition), as these appointments would not practically allow time for an intervention. This step ensured that the dataset was consistent with our definition of a no-show and suitable for building a ML model which would be used in practice.

The final transformed dataset contained 38 141 appointments from 21 936 patients over the 7-year period. The average no-show rate across this dataset was 16.6%, although, as shown in Figure 2, this rate fluctuated between 12% and 22% during the study period. The final set of variables available for modeling is summarized in Table 1.

### Model building and evaluation

**Figure 3.**
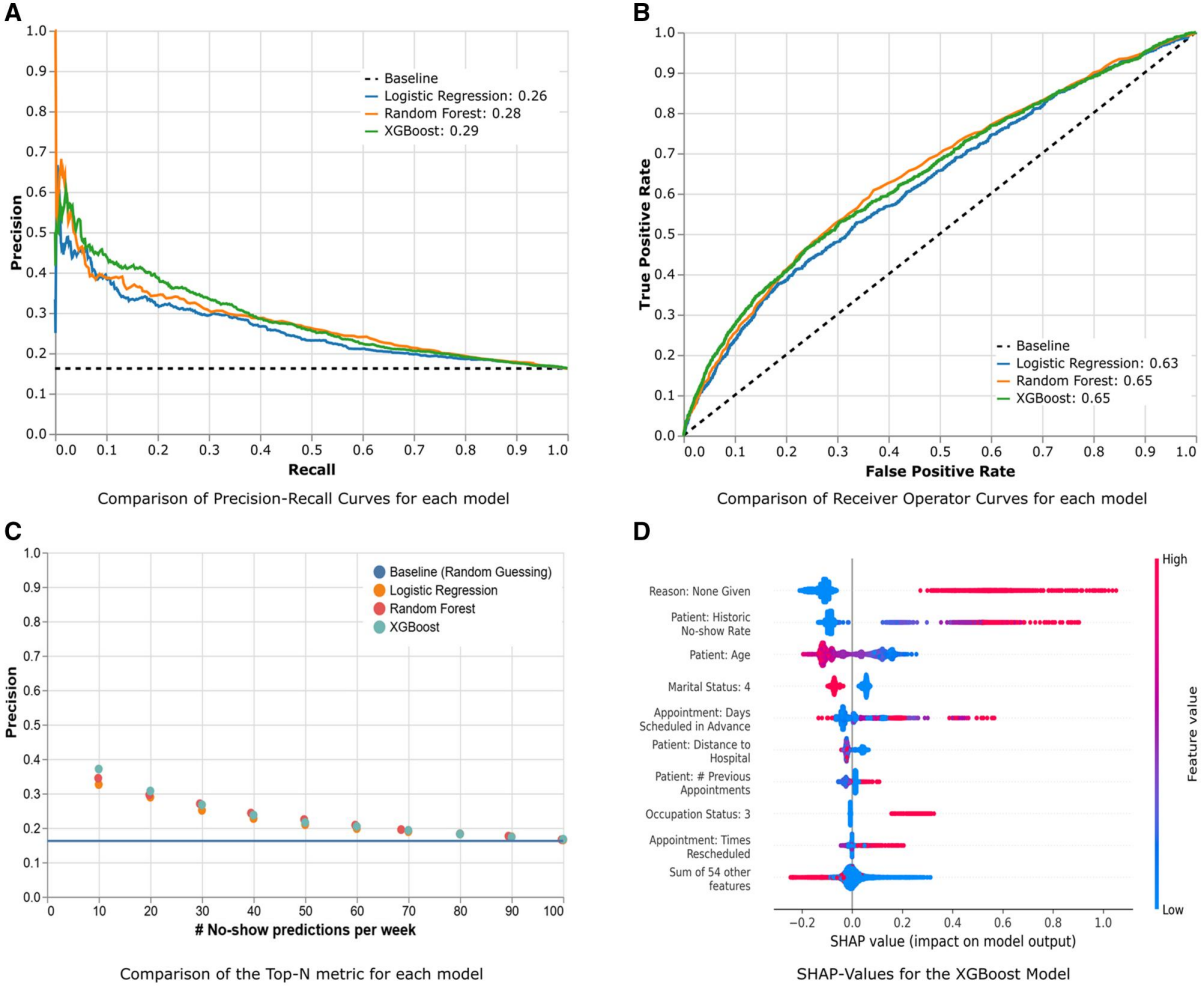
Results from predictive models. (A) Comparison of precision-recall curves for each model. (B) Comparison of receiver operator curves for each model. (C) Comparison of the top-N metric for each model. (D) SHAP-values for the XGBoost model.

After training each model on the training data, we evaluated its performance on the hold-out validation set. Each model showed an improvement over the baseline of random guessing, with XGBoost and Random Forest demonstrating particularly strong performance. As shown in Figure 3A, XGBoost achieved the highest average AUPRC at 0.29, closely followed by Random Forest at 0.28, while in terms of AUROC, Figure 3B shows that XGBoost and Random Forest performed equally well (AUROC: 0.65). Logistic Regression, while surpassing the baseline, had comparatively lower performance with an AUPRC of 0.26 and an AUROC of 0.63.

Figure 3C presents the Top-N precision, showing model performance as a function of the number of weekly “no-show” predictions. All models outperformed random guessing (baseline: 0.17), with XGBoost performing best, achieving precision scores of 0.38 and 0.31 for the Top 10 and 20 predictions, respectively. This indicates that targeting the top 10 high-risk patients weekly could identify nearly 4 true no-shows, supporting the model’s potential for guiding targeted interventions.

To enhance interpretability of the best-performing model, we calculated SHAP values, which quantify the contribution of each feature to the predictions.^18^ As shown in the SHAP plot in Figure 3D, the *Historic No-show Rate* feature is a strong predictor, with higher past no-show rates (represented in red) increasing the predicted likelihood of a no-show for the upcoming appointment. The feature *Reason*, specifically with the value “None Given” also has a significant impact on the predictions, suggesting that patients without a recorded reason for their appointment are at higher risk of no-show. Additional important predictors included patient age, marital status, and the number of days between scheduling and the appointment date. Conversely, features such as *Day of the Week*, *Gender*, and *Modality of Appointment* were not among the top predictors, indicating a comparatively minor impact on the model’s predictions.

### Silent live test

We conducted the SLT over a 13-month period, from February 2022 to March 2023, further validating our best-performing model (XGBoost) on an additional 5882 appointments. On this dataset, the model achieved an AUROC of 0.61 and an AUPRC of 0.24, a reduction in performance when compared to the validation data performance (AUROC: 0.65, AUPRC: 0.29).

**Figure 4.**
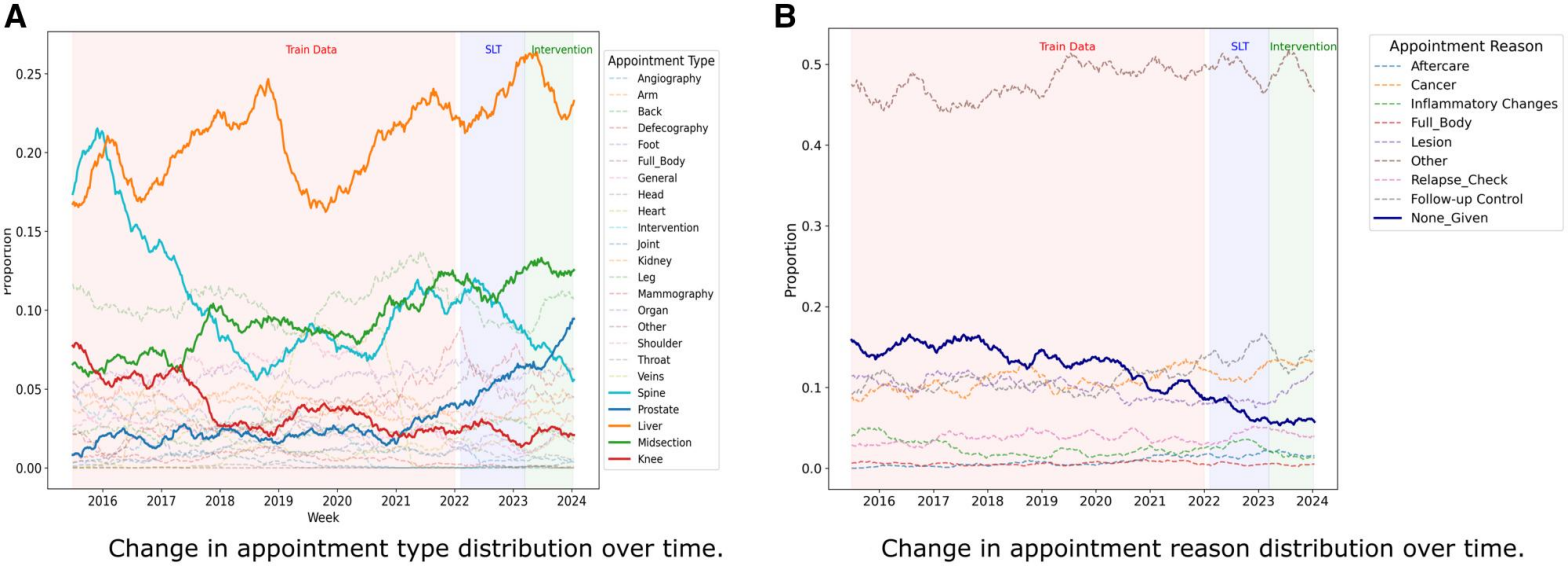
Shifts in appointment data distributions over time, illustrating data drift between training and deployment periods. (A) Change in appointment type distribution over time. (B) Change in appointment reason distribution over time.

This drop in performance can be explained in part by data drift—that is, changes in a feature’s distribution over time. Figure 4 illustrates these shifts, showing trends in appointment types (Figure 4A) and reasons (Figure 4B), with selected categories highlighted for emphasis. For example, prostate appointments represented just 2%-3% of all appointments during the 2015-2021 training period, but steadily increased to 5% during the SLT and nearly 10% by the end of the intervention. Conversely, the proportion of knee appointments and spine appointments declined over time, while liver appointments exhibited high variability throughout. Appointment reasons also shifted over time—the label ‘None Given’ accounted for approximately 13%-15% of appointments between 2015 and 2019, but fell to 5%-6% by the intervention period. This shift is particularly noteworthy, as this feature was the most predictive variable in the model according to SHAP analysis.

### Intervention

The intervention study ran for nine months in 2023, from March 13th to December 14th. During this period, scheduling staff contacted a subset of patients identified as high-risk by our model and reminded them of their upcoming appointment. A power analysis indicated that a minimum sample size of 200 appointments was required to detect an improvement of one fewer no-show per week with at least 80% power. In total, 274 patients were contacted, of whom 158 (57.7%) answered the call, while the remaining 116 (42.3%) did not. The control group consisted of 460 patients. Summary results are presented in Table 2, and results stratified by risk quartiles are shown in Table 3. Overall, the phone call trial showed no significant reduction in the no-show rate between the control (22.0%) and intervention (21.9%) groups (*P-*value: .48), as shown in Table 2. However, within the intervention group, patients who answered the call had a lower no-show rate (18.4%) than those who were not reached (26.7%), a relative difference in no-show rate of 31%.

**Table 2.** Intervention study results.

Group	Sub group	# Appointments (# no-shows)	No-show rate
Control		460 (101)	22.0%
Intervention	All	274 (60)	21.9%
	Not Reached	116 (31)	26.7%
	Reached	158 (29)	18.4%

**Table 3.** Comparison of control and intervention groups across quartiles of predicted no-show risk, within the high-risk cohort. The 1st quartile represents the highest-risk patients (top 25%), while the 4th quartile represents the lowest-risk patients (bottom 25%).

1st Quartile (highest risk)			
Group	Sub group	# Appointments (# no-shows)	No-show rate
Control		120 (34)	28.3%
Intervention	All	63 (19)	30.2%
	Not reached	26 (9)	34.6%
	Reached	37 (10)	27.0%

2nd Quartile			
Group	Sub group	# Appointments (# no-shows)	No-show rate
Control		108 (24)	22.2%
Intervention	All	75 (17)	22.7%
	Not reached	36 (9)	25.0%
	reached	39 (8)	20.5%

3rd Quartile			
Group	Sub group	# Appointments (# no-shows)	No-show rate
Control		111 (20)	18.0%
Intervention	All	73 (15)	20.5%
	Not Reached	30 (8)	26.7%
	Reached	43 (7)	16.3%

4th Quartile (lowest risk)			
Group	Sub group	# Appointments (# no-shows)	No-show rate
Control		121 (23)	19.0%
Intervention	All	62 (9)	14.5%
	Not Reached	24 (5)	20.8%
	Reached	38 (4)	10.5%

To explore these results further, we analyzed the effect of the intervention across subgroups of the study population, divided into quartiles based on the predicted no-show probability. In the top three quartiles (ie, higher-risk patients), no significant differences were observed between the intervention and control groups. In the lowest-risk quartile, the intervention group had a slightly lower no-show rate (14.5%) compared to the control group (19.0%), though this difference was not statistically significant. Across the full study population and within each quartile, patients who answered the reminder call had consistently lower no-show rates than both those who were not reached and those in the control group. In contrast, patients who were called but were not reached exhibited the highest no-show rates across all groups.

### Application user interface

A demo web application was built using React (v18.2.0). The application was a proof-of-concept and thus had no backend connection—instead, data were generated randomly and displayed in the application. The application has two main views: the first displayed the daily appointment schedule (see Appendix A, Figure SA1), with appointments color-coded by no-show risk (blue = low, yellow = medium, red = high). The second view lists all appointments scheduled for the next week, ordered by predicted no-show probability. We evaluated the user interface using the QUIS with three members of the scheduling staff. Each member rated the application independently, providing an overall score as well as scores for specific qualities of the interface, rated on a scale from 0 to 9. The results (see Appendix A, Table SA1) showed a mean score of 7.5 (min: 6.33, max: 8.33) across 8 different categories.

## Discussion

This study set out to reduce no-show rates for MRI appointments in a hospital radiology department through a comprehensive project, by developing, validating, and deploying a ML model to inform targeted patient phone call reminders. The model performed well in identifying high-risk patients using routinely collected scheduling data and was successfully used in a real-world intervention. However, while the predictive performance of our model was strong, translating this into a measurable improvement in attendance proved more difficult.

### Learnings from modeling

Our results demonstrate that ML models can predict no-shows more accurately than baseline measures, with XGBoost performing best (AUROC: 0.65; AUPRC: 0.29; Top-10 Precision: 0.38). These findings suggest that predictive modeling could serve as a valuable tool in addressing operational inefficiencies in hospitals. Interpreting the importance of features through SHAP values further showed that past no-show behavior and appointment reasons were among the strongest predictors.

However, our “silent live test” highlighted a key challenge: data drift, where shifts in data distribution over time resulted in a degradation of model performance. This shows the importance of continuous monitoring and updating of predictive models in order to maintain their robustness. Future research should explore regular model updates with new data, as well as more robust modeling methods, to ensure long-term effectiveness.

### Learnings from the intervention

Although the modeling phase showed the potential to predict no-shows effectively, our intervention study revealed that reducing actual no-show rates in practice is more complex than simply achieving strong predictive accuracy. One likely factor in this outcome was the limited reach of the intervention itself—42.3% of patients in the intervention group were not reached by the reminder calls. Among those reached, the no-show rate was 18.4% compared to 26.7% for those who were not reached—a relative reduction of 31%. This suggests that a patient’s responsiveness to a phone call may itself serve as a proxy for their likelihood to attend.

While the intervention did not significantly reduce overall no-show rates, there were promising signs in certain subgroups. In the lowest quartile of high-risk patients, the intervention showed a slight, albeit statistically insignificant, improvement, suggesting that future interventions could benefit from a more targeted approach. One potential strategy could involve predicting not only the likelihood of a no-show, but also the likelihood of a positive response to an intervention. Such a dual-prediction strategy would allow for better resource allocation, prioritizing patients who are both at risk of missing their appointments and likely to respond to reminders.

### Overall learnings and limitations

This study emphasizes the need for a thorough approach to address the issue of no-show appointments in healthcare. While predictive models can help identify patients at higher risk of missing appointments, the success of interventions will require a deeper understanding of the reasons behind no-shows and the development of targeted strategies to address these reasons. In addition to this, expectations for intervention strategies should be realistic, since there is likely a theoretical limit to how much the number of no-shows can be reduced, as some patients will inevitably miss appointments due to unforeseen circumstances. Therefore, an effective approach must be holistic, combining predictive insights with systemic measures such as more flexible scheduling for patients, varied communication methods, and a balance of incentives and penalties. Our web-based tool shows how predictions could be integrated into everyday workflows, but further development and evaluation in operational settings will be needed before such systems can be implemented at scale.

This study also has limitations. The model was built on a relatively narrow feature set within a single-center study using data from two MRI machines, which may limit generalizability. Model performance degraded over time due in part to data drift, showing the need for continuous monitoring and model retraining in practice. Finally, although the intervention was adequately statistically powered overall, analyses of subgroups (eg, by risk quartile) were based on smaller numbers and therefore limited in statistical power.

The intervention was kept deliberately simple: outcomes were recorded only as “reached” or “not reached,” without accounting for voicemails, invalid numbers, or third-party answers, and no standardized script was used. The calls themselves were also somewhat ad hoc in their execution. Unanswered calls were not retried, and patient responses beyond the binary outcome were not documented, limiting both the reach of the intervention and the detail in which its effects can be analyzed.

### Comparison with prior work

Previous studies on no-shows in radiology departments have focused primarily on retrospective model development and internal validation, identifying predictors such as modality, lead time, and patient demographics.^19–22^ Systematic reviews highlight past attendance, socioeconomic status, and scheduling delay as key factors across specialties.^23^^,^^24^ However, few studies have evaluated how these models and their predictions can be deployed to inform targeted interventions in real-world clinical workflows. Our study contributes to this gap by combining model development, silent live testing, and a prospective intervention within an operational radiology department. This end-to-end approach offers a more comprehensive view of what is required to translate predictive models into strategies for reducing no-show rates.

### Future work

Future work should focus on enhancing predictive performance and improving the effectiveness of interventions. Incorporating additional data sources—such as patient communication history, transportation access, or weather forecasts, alongside more advanced ML techniques (such as deep learning and transfer learning) could improve prediction accuracy. To ensure robustness over time, predictive models should also be continuously updated with recent data to mitigate the effects of data drift.

Developing and testing new intervention strategies which target specific patient subgroups or use a broader range of communication methods could improve engagement. Future efforts could also include not just a prediction of which patients are likely to no-show, but also which patients would respond well to an intervention.

Further research should also investigate the underlying reasons behind nonattendance and how best to incentivize patients to keep their appointments. A valuable extension of this study would involve following up with patients who were contacted but still did not attend, in order to better understand barriers to attendance.

Finally, a software application could be integrated into hospital scheduling systems to track prediction accuracy and intervention outcomes in real time. Based on the positive staff feedback, the tool shows promise for adoption, but further development toward a production-ready version should be guided closely by end-user input.

## Conclusion

In this study, we developed and evaluated ML models to predict patient no-shows for radiology appointments at the University Hospital of Zurich. XGBoost performed best, but our “silent live test” revealed performance degradation on recent data due to data drift, highlighting the need for continuous monitoring and model updating in deployment. A subsequent phone call intervention for high-risk patients did not significantly reduce overall no-show rates. However, it did reveal that patients who answered the calls were less likely to miss their appointments, suggesting that targeted interventions and direct communication with patients could be effective strategies for reducing no-shows. Ultimately, this study highlights the potential of ML models in predicting no-shows, while also emphasizing the importance of robust pipelines, adaptive models, and more advanced intervention strategies. Future research should focus on regularly updating models, exploring alternative intervention methods, and tailoring approaches to specific patient subgroups.

## Supplementary Material

ooag151_Supplementary_Data

## Data Availability

The data used in this study can be requested from the authors’ institution upon reasonable request, for noncommercial research and validation purposes. Access would be subject to a data transfer agreement and approval from the relevant ethical and data governance boards.

## References

[1] Curran J , Halpert R, Straatman A. Patient “no-shows”–a costly problem. Radiol Manage. 1989;11:20-23. 10291948

[2] Harvey HB , Liu C, Ai J, et al Predicting no-shows in radiology using regression modeling of data available in the electronic medical record. J Am Coll Radiol. 2017;14:1303-1309. 28673777 10.1016/j.jacr.2017.05.007

[3] Berg BP , Murr M, Chermak D, et al Estimating the cost of no-shows and evaluating the effects of mitigation strategies. Med Decis Making. 2013;33:976-985. 23515215 10.1177/0272989X13478194 PMC4153419

[4] Rosenbaum JI , Mieloszyk RJ, Hall CS, Hippe DS, Gunn ML, Bhargava P. Understanding why patients no-show: observations of 2.9 million outpatient imaging visits over 16 years. J Am Coll Radiol. 2018;15:944-950. 29755001 10.1016/j.jacr.2018.03.053

[5] Kheirkhah P , Feng Q, Travis LM, Tavakoli-Tabasi S, Sharafkhaneh A. Prevalence, predictors and economic consequences of no-shows. *BMC Health Serv Res.* 2016;16:13. 10.1186/s12913-015-1243-z PMC4714455 26769153

[6] Xakellis G , Bennett A. Improving clinic efficiency of a family medicine teaching clinic. Family Medicine-Kansas City. 2001;33:533-538. 11456246

[7] Moore CG , Wilson-Witherspoon P, Probst JC. Time and money: effects of no-shows at a family practice residency clinic. Family Medicine-Kansas City. 2001;33:522-527. 11456244

[8] Taber DJ , Fleming JN, Fominaya CE, et al The impact of health care appointment non-adherence on graft outcomes in kidney transplantation. Am J Nephrol. 2017;45:91-98. 27907919 10.1159/000453554 PMC5214896

[9] Schmalzried HD , Liszak J. A model program to reduce patient failure to keep scheduled medical appointments. J Community Health. 2012;37:715-718. 22057423 10.1007/s10900-011-9505-0

[10] Deina C , Fogliatto FS, da Silveira GJC, Anzanello MJ. Decision analysis framework for predicting no-shows to appointments using machine learning algorithms. BMC Health Serv Res. 2024;24:37. 10.1186/s12913-023-10418-6. 38183029 PMC10770919

[11] Liu D , Shin WY, Sprecher E, et al Machine learning approaches to predicting no-shows in pediatric medical appointments. NPJ Digit Med. 2022;5:50. 10.1038/s41746-022-00594-w. 35444260 PMC9021231

[12] Oikonomidi T , Norman G, McGarrigle L, Stokes J, van der Veer SN, Dowding D. Predictive model-based interventions to reduce outpatient no-shows: a rapid systematic review. Journal of the American Medical Informatics Association. 2023;30:559-569. 10.1093/jamia/ocac242. 36508503 PMC9933067

[13] Chong LR , Tsai KT, Lee LL, Foo SG, Chang PC. Artificial intelligence predictive analytics in the management of outpatient MRI appointment no-shows. American Journal of Roentgenology. 2020;215:1155-1162. 32901567 10.2214/AJR.19.22594

[14] Elvira C , Ochoa A, Gonzalvez JC, Mochón F. Machine-learning-based no show prediction in outpatient visits. International Journal of Interactive Multimedia & Artificial Intelligence. 2018;4:29-34.

[15] Mieloszyk RJ , Rosenbaum JI, Bhargava P, Hall CS. Predictive modeling to identify scheduled radiology appointments resulting in non-attendance in a hospital setting. In: 2017 39th Annual International Conference of the IEEE Engineering in Medicine and Biology Society (EMBC). IEEE 2017: 2618-21. 10.1109/EMBC.2017.8037394 29060436

[16] Mieloszyk RJ , Rosenbaum JI, Hall CS, Hippe DS, Gunn ML, Bhargava P. Environmental factors predictive of no-show visits in radiology: observations of three million outpatient imaging visits over 16 years. Journal of the American College of Radiology. 2019;16:554-559. 30947887 10.1016/j.jacr.2018.12.046

[17] Chin J , Diehl V, Norman K. Development of an Instrument Measuring User Satisfaction of the Human-Computer Interface. In: *Proceedings of the SIGCHI Conference on Human Factors in Computing Systems (CHI '88)*. Association for Computing Machinery; 1988 01.

[18] Lundberg SM, Lee SI. A unified approach to interpreting model predictions. In: *Proceedings of the 31st International Conference on Neural Information Processing Systems (NIPS '17)*. Curran Associates Inc.; 2017:4768-4777.

[19] Shah P , Shah M, Schwartz S, Farrow M, Lu A. Machine learning approaches to predict no-shows in pediatric outpatient clinics. Stud Health Technol Inform. 2023;302:273-277.

[20] Daggy J , Lawley M, Willis D, et al Using no-show modeling to improve clinic performance. Health Informatics J. 2010;16:246-259. 21216805 10.1177/1460458210380521

[21] Harvey HB , Liu C, Ai J, et al Predicting no-shows in radiology using regression modeling of data available in the electronic medical record. Journal of the American College of Radiology. 2017;14:1303-1309. 28673777 10.1016/j.jacr.2017.05.007

[22] Yassaie H , Nandurkar D, Scott K, Gorelik A. No-show rates in radiology: analysis of over 2.9 million appointments over 16 years. Journal of the American College of Radiology. 2018;15:662-669.

[23] Huang Y , Hanauer DA. Patient no-show prediction: a systematic literature review. J Healthc Inform Res. 2018;2:172-193.

[24] Langarizadeh M , Tavakol K, Ahmadi S. Predicting patient no-shows in outpatient appointments using machine learning techniques. South Journal of Health. 2023;5:24-31.

