# Supplementary material for: From prediction to practice: machine learning models and real-world interventions for magnetic resonance imaging appointment no-shows in a hospital setting
Source: JAMIA Open. 2026 Aug 4;9(4):ooag151. doi: 10.1093/jamiaopen/ooag151 (PMC13437018; doi:10.1093/jamiaopen/ooag151)
Supplement: Supplementary file 1 [file ooag151_supplementary_data.pdf]

Appendix A Demo Application Results

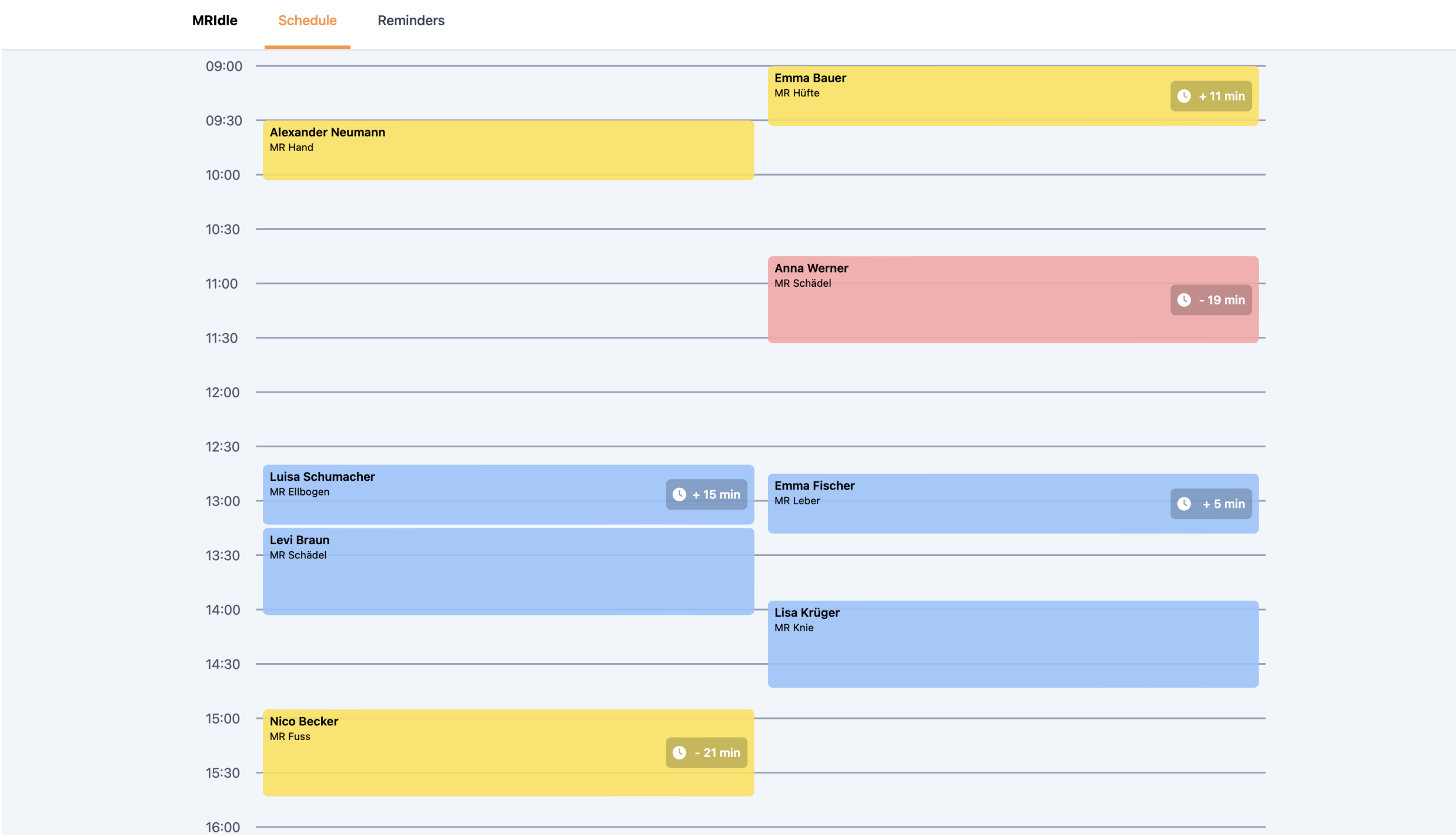

Figure A1: The demo application, showing our implementation of the model's predictions into everyday practice. Appointments with a predicted risk of a no-show are marked in yellow (medium risk) or red (high risk). Additionally, the application displays if an appointment is predicted to take longer or shorter than the scheduled time slot, which could be an additional feature in future research. Any names and other patient data are randomly generated.

**Feature**	**Score 0**	**Score 9**	**Rater 1**	**Rater 2**	**Rater 3**	**Mean**
Overall impression on the application	frustrating	satisfying	8	5	8	7.0
Organization of information on screen and readability	confusing	very clear	8	7	8	7.7
Sequence of screens	confusing	very clear	8	7	8	7.7
Terminology is related to the task you are doing	never	always	5	8	6	6.3
System speed	too slow	fast enough	8	7	9	8.0
System is intuitive to use	difficult	easy	8	6	9	7.7
Use of colors	poor	good	8	8	9	8.3
System feedback	poor	good	8	7	7	7.3

Table A1: Questionnaire results
